# Learning and generalization of repetition-based rules in autism

**DOI:** 10.1007/s00426-022-01761-0

**Published:** 2022-11-09

**Authors:** Roberta Bettoni, Margaret Addabbo, Chiara Ghidina, Chiara Pezzana, David Vagni, Chiara Turati, Hermann Bulf

**Affiliations:** 1 Department of Psychology, University of Milano-Bicocca, Milan, Italy; 2 NeuroMi, Milan Center for Neuroscience, Milan, Italy; 3 Associazione per l’Autismo E. Micheli Onlus, Novara, Italy; 4 CNR-IRIB Institute for Biomedical Research and Innovation National Research Council of Italy, Mortellec, Messina Italy

## Abstract

Rule Learning (RL) allows us to extract and generalize high-order rules from a sequence of elements. Despite the critical role of RL in the acquisition of linguistic and social abilities, no study has investigated RL processes in Autism Spectrum Disorder (ASD). Here, we investigated RL in high-functioning autistic adolescents with ASD, examining whether their ability to extract and generalize rules from a sequence of visual elements is affected by the social vs. non-social nature of the stimulus and by visual working memory (WM). Using a forced-choice paradigm, ASD adolescents and typically developing (TD) peers were tested for their ability to detect and generalize high-order, repetition-based rules from visual sequences of simple non-social stimuli (shapes), complex non-social stimuli (inverted faces), and social stimuli (upright face). Both ASD and TD adolescents were able to generalize the rule they had learned to new stimuli, and their ability was modulated by the social nature of the stimuli and the complexity of the rule. Moreover, an association between RL and WM was found in the ASD, but not TD group, suggesting that ASD might have used additional or alternative strategies that relied on visual WM resources.

## Introduction

Unspoken rules govern language and many social events. The ability to grasp unspoken regularities plays an adaptive role in responding appropriately to one’s social environment. Mastery of these rules can be a struggle in some developmental disabilities, such as the Autism Spectrum Disorder (ASD, Jones et al., 2013; Ullman, 2016). For example, ASD individuals often show difficulties in learning rules underpinning social interactions (Bottini, 2018; Jones et al., 2013) and in applying this knowledge to different social situations (e.g., Ozonoff & Miller, 1995). The deficit in the ability to efficiently identify relationships across events may not allow individuals with ASD to form a coherent representation of the world, perceiving it, instead, as a complex and chaotic environment (Klinger et al., 2007; Sinha et al., 2014).

The present study aimed to investigate the ability to extract and generalize abstract rules in ASD individuals and to assess whether this ability is affected by the presence of social signals. Several studies have investigated prototypical category learning in ASD, showing a deficit in creating a mental summary representation of multiple experienced stimuli that go together in a category (see Klinger & Dawson, 2001; Vanpaemel & Bayer, 2021). However, the ability to detect regularities is not only related to specific characteristics of the stimuli across events, but also to the temporal relationship between elements in a sequence. For example, many studies have focused on a learning process named statistical learning (SL) that allows to rapidly extract and use of structured sequential information, such as the statistical relationships between elements (Saffran et al., 1999). Studies investigating SL in ASD population have shown that, despite comparable behavioral performances in detecting statistical regularities embedded in a sequence of syllables (Mayo & Eigsti 2012), children with and without autism show different neural activity patterns in learning statistical associations (Scott-Van Zeeland et al. 2010). This evidence is further supported by a recent study showing a different pattern of electrophysiological response in tracking visual statistical regularities in ASD children (Jeste et al., 2015) and in infants at high-risk of developing ASD (Marin et al., 2020) relative to typically developing children and infants.

Yet, the ASD disadvantage to elaborate sequential information may not be limited to the detection of simple statistical re-occurrences. Such a disadvantage may extend to more complex learning processes still based on sequential processing, such as Rule Learning (RL), that is defined as the ability to detect repetition-based rules from sequences of stimuli and to generalize them to a new set of stimuli, going beyond the perceptual characteristics of the items that give rise to the to-be-learned rules (Marcus et al., 1999). More specifically, RL relies on the ability to keep track of the invariant positional relation of an item within a sequence and to generalize it beyond the finite bounds of the stimuli information. RL was first demonstrated for speech sequences in early infancy (Marcus et al., 1999). After being familiarized with a sequence of syllables that followed a high-order, repetition-based rule (ABB, AAB, ABA), preverbal infants were able to extract the rule and generalize it to a new set of syllables (Marcus et al., 1999). From early development, RL has also been demonstrated in the visual modality, even though it appears constrained by several factors imposed by limits in attention and memory (e.g., Schonberg et al., 2018) and by the direction of spatial information with which the sequences of the stimuli are delivered (Bulf et al., 2017). Many studies have also demonstrated that, starting from the first months of age, RL involves a broad and abstract representation of the to-be-learned rule (Rabagliati, Ferguson, Lew-Williams, 2019) and it allows a transfer of learning across domains (Bulf et al., 2022; Marcus et al., 2007) and modalities (Bulf et al., 2021). While RL has mainly been investigated at the early stages of development, it remains available in adulthood. Investigating the neural correlates of auditory abstract rules acquisition, i.e., ABB and ABA, differences in the N400—an electrophysiological component related to rule error detection—have been observed in adults, indicating that they are sensitive to the inconsistency between adjacent and non-adjacent abstract rules (Sun et al., 2012).

More recently, it has been proposed that early abilities to identify and generalize abstract rules are at the basis of the ability to learn from others in linguistic and communicative domains (Bettoni et al., 2020; Endress & Bonatti, 2016), and it is one of the building blocks to discover, later in development, increasingly complex abstract structures characterizing social situations (Lieberman, 2000). For example, early abilities to extract regularities across temporal events play a critical role in the development of social understanding (Hunnius & Bekkering, 2014; Ruffman et al., 2012) and the functioning of RL is closely related to the ability to process social signals (Bulf et al., 2015; Ferguson & Lew-Williams, 2016; Rabagliati et al., 2019). This result is in line with evidence that RL is modulated by the presence vs. absence of social stimuli provided by an interaction between two agents (Ferguson & Lew-Williams, 2016) or by social touch (Lew-Williams et al., 2019).

Overall, this evidence indicates that RL plays a pivotal role in the development of high-level cognitive abilities, such as communicative skills, and that its functioning is related to meaningful social signals. Thus, the investigation of RL in the ASD population appears particularly relevant, as the ability to extract and generalize high-order rules might resemble those needed to face the learning challenge of creating an abstract representation of rules from communicative and social events and generalizing it to different circumstances (Klinger et al., 2007).

The present study aimed to investigate RL abilities in ASD individuals and to assess whether their ability to extract and generalize an abstract rule is affected by the presence of social signals. To this end, we exposed 14–18-year-old ASD adolescents without intellectual disabilities (IQ > 75, high functioning individuals) and a control group of typically developing (TD) peers to sequences of visual elements organized into repetition-based rules (i.e., ABB and ABA). After being exposed to triplets of visual elements following ABA or ABB rules and sequentially presented from left to right, adolescents were presented with a triplet instantiated by new items following new or familiar rules. The participants were verbally instructed to identify whether the sequences of visual items were congruent (same rule) or incongruent (different rule) from the one presented in the learning phase. The triplets were presented sequentially from left to right since RL seems modulated by the spatial presentation of the stimuli, with the advantage of recognizing the rules when they are presented from left to right (Bulf et al., 2017).

Many studies have demonstrated that sequential and implicit learning are spared in ASD when behavioral tasks are used (Foti et al., 2015; but see Bettoni et al., 2021, and Jeste et al., 2015, for the role of implicit vs. explicit sequential learning in ASD). In our task, we instructed both groups of adolescents to detect whether the order of the stimuli within the sequences was the same or not, giving a cue to extract the rule embedded in the triplet of items, while ignoring the change in the item identity. Starting from this evidence, we might expect intact RL abilities in both ASD and TD groups, with a between group difference in learning abstract rules from social vs. non-social stimuli, i.e., upright vs. inverted faces. Indeed, converging evidence showed atypical face processing in ASD (Dawson et al., 2005; Webb et al., 2017). For example, some studies have reported that the face inversion effect is reduced in ASD (McPartland et al., 2004; Vettori et al., 2019). This might reflect a feature-based strategy during face recognition, which might be useful in recognizing inverted faces, but is not fully efficient in processing upright faces (Rossion, 2009) and might explain some anomalies in processing upright faces in ASD (Webb et al., 2017). Following this line of reasoning, we might expect the worst performances in the ASD group in generalizing rules from upright faces compared to inverted faces and geometrical shapes.

Lastly, we investigated whether individual differences in ASD vs. TD working memory (WM) are correlated to visual RL abilities. Indeed, when individuals are faced with sequential, temporal information, they must also keep track of the items in the sequence over time and hold information about the temporal succession of the element in the sequence, a process that strongly depends on WM (e.g., van Abswoude et al. 2020). WM is considered a critical factor in mediating sequence learning in general (Abrahamse et al., 2014) and RL in particular, since it implies the encoding of visual elements, the identification of rules across time, and the comparison between sequences seen in the learning and test phases (Bulf et al., 2017). To this aim, visual WM capacities were measured through the Corsi Block Tapping-test (Mammarella 2008), and associations between WM scores and RL abilities were examined in ASD and TD peers. Remarkably, evidence shows that there are deficits in both phonological and visual WM in the ASD population (for a review, see Habib et al., 2019; Unsworth et al., 2005). We hypothesized to find a strong correlation between WM scores and RL measures only in the ASD group. Indeed, if ASD adolescents succeed in the RL task, we expect them to show more difficulties in RL by using different strategies compared to the TD group.

## Materials and methods

### Participants

Eighteen typically developing adolescents (TD group) and 18 age-matched adolescents with a diagnosis of autism spectrum disorder (ASD group) participated in the study. The sample size was estimated based on an a-priori Power Analysis for a within-between repeated-measures Analysis of Variance (ANOVA) (Faul et al., 2007). To obtain a medium effect size of 0.20 with *α* = 0.05 and power = 0.90, the total sample size was *N* = 36. The two groups were matched for chronological age, *t*(34) = 1.054, *p* = 0.299. Diagnosis of autism was established by expert clinicians through the DSM-5 diagnostic criteria, the Autism Diagnostic Observation Scale (ADOS) (Lord et al., 1989), and the Childhood Autism Rating Scale (CARS; Schopler, Reichler, & Renner, 2010).

Inclusion criteria in the ASD group were: (i) IQ within the normal range, with a score greater than 75 on standardized intelligence tests, such as the Wechsler Intelligence Scale for Children-Revised (WISC-R; Wechsler, 1994; *N* = 17) or the Leiter-R scale (Roid & Miller, 1997; *N* = 1)); (ii) absence of comorbidity with mood and anxiety disorders, and attention deficit hyperactivity disorder based on DSM-5 criteria (American Psychiatric Association, 2013). Importantly, the ASD participants did not receive medical treatments at the time of the experiment. The cognitive level of the TD group was assessed with three verbal (vocabulary, similarities, and digit span) and two performance (block design and picture completion) subtests of the WISC-R (Wechsler, 1994), with norms available for the Italian populations (Orsini & Picone, 2006). All participants of the TD group obtained a score within the normal range on each subtest of the WISC-R (Italian version; Orsini & Laicardi, 1993; see Table 1). In addition, visual WM capacities were assessed in both groups through the Corsi Block Tapping-test (ASD raw-score of the visual span range = 4–8; *Mean* raw score = 5.83, *SD* = 1.04; TD raw-score of the visual span range = 4–7, *Mean* raw score = 6.22, *SD* = 0.808; Mammarella et al., 2008; Table 1). After completion of the RL testing session, data from additional three participants were excluded from the final analyses because their accuracy (*n* = 1 in ASD) or reaction times (*n* = 1 in ASD and *n* = 1 in TD) were 2.5 DS below the mean. ASD participants were recruited at the ‘Associazione per l'Autismo E. Micheli’ based in Novara (Italy) and the TD group from several public schools of Novara (Italy). Informed consent was obtained from all the participants and their parents. The procedure was approved by the Ethics Committee of the University of Milano-Bicocca, following principles expressed in the Declaration of Helsinki (World Medical Association, 2013).

**Table 1:** Descriptive statistics of the TD and ASD group. The sub-tests of Wisc-R are reported in weighted scores, whereas the Corsi Block Tapping-test scores are reported in raw scores and z-scores

	TD group (*N* = 18, male = 13)		ASD group (*N* = 18, male = 18)	
Variables	Mean	SD	Mean	SD
Age (years)	16.85	1.19	16.38	1.50
Total IQ	–	–	101.82	15.68
Digit-span sub-test	11.94	1.76	–	–
Vocabulary sub-test	11.17	1.79	–	–
Similarity sub-test	11.83	1.65	–	–
Picture completion sub-test	11.83	2.87	–	–
Block design sub-test	12.83	2.55	–	–
Corsi Block Tapping-test (raw score)	6.22	0.81	5.83	1.04
Corsi Block Tapping-test (z score)	1.20	0.93	0.65	1.22

### Apparatus and stimuli

**Fig. 1:**
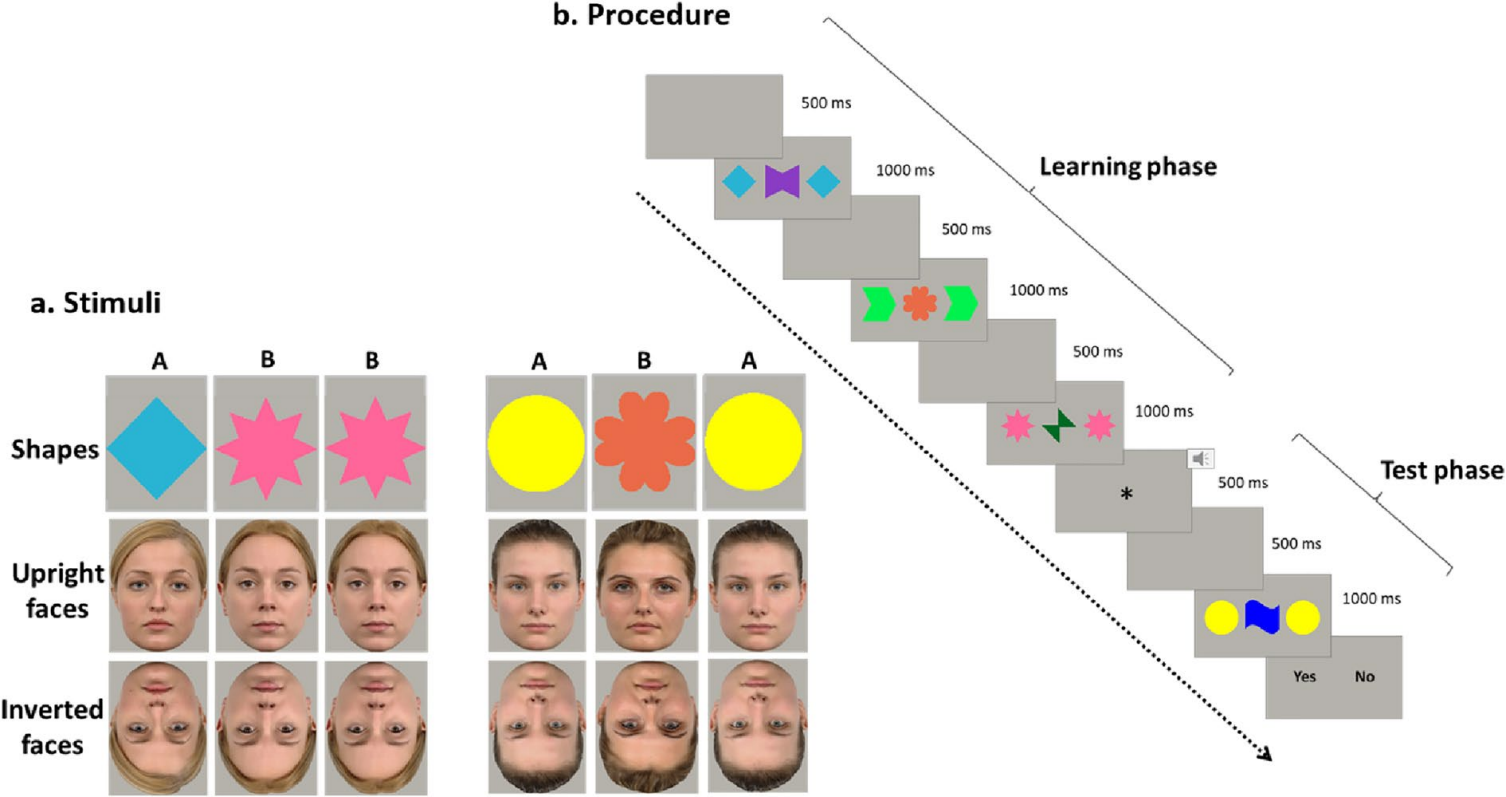
**a** Examples of stimuli for each of the three conditions used in the RL task (geometrical shapes, upright face, inverted faces) and for each rule (ABB, ABA). **b** Example of a trial structure

The testing session was conducted in a quiet and well-lit room, approximately 60 cm from a 15″ computer monitor (1280 × 800 pixels). Stimulus presentation was performed with E-Prime 2 (Psychology Software Tools, Inc.). Stimuli consisted of 16 geometrical shapes, 16 upright faces and 16 inverted faces, embedded in a virtual square subtending a visual angle of 10° × 10°. Shapes stimuli were 16 geometrical objects of different colors. Upright faces consisted of photographs of 16 Caucasian women with a neutral facial expression selected from a validated database, i.e., Radboud Faces Database (Langner et al., 2010). Images were cropped so that some external features (i.e., ears and hair) were still visible. Face images were turned upside-down (180° rotation) to create the inverted face stimuli set (Fig. 1).

### Procedure

#### Rule learning task

Each participant took part in three ~ 20 min experimental sessions in which RL abilities were assessed using: (i) social stimuli (i.e., faces); (ii) non-social stimuli with high complexity (i.e., inverted faces); (iii) non-social stimuli with low complexity (i.e., shapes). QueryThe three experimental sessions were administered separately on a weekly basis, and the order of presentation was counterbalanced to mitigate the effects of interference among tasks. IQ information for the ASD group was obtained from the clinical record, while general cognitive ability (WISC-R) for the TD group was evaluated during the experimental sessions. Moreover, visual WM was assessed in one of the three experimental sessions for both groups using the Corsi Block Tapping-test (Mammarella et al., 2008).

Participants were instructed to respond as fast as possible, choosing whether the sequence of elements was congruent (same rule) or incongruent (different rule) from the one presented in the learning phase. In particular, the instruction was: “In this test, you will see 3 shapes that follow one another in a particular order. Sometimes the order can be the same, sometimes not. You will have to tell me if the order you see is the same or not, as quickly and accurately as possible, by pressing the green button for yes or red button for no”. In each experimental condition (shape, upright face, and inverted face), the visual RL was composed of 72 trials, and each trial consisted of a learning and a test phase (Fig. 1). Before the experimental session, participants were presented with a training phase of 4 trials to familiarize the participant with the procedure. The participants’ responses recorded during the training phase were not included in the analysis. As soon as the training phase was over, the experimental session began.

During the learning phase of each trial, participants were presented with three different triplets arranged with an ABB (less complex rule, adjacent repetition; *N* = 30 trials) or an ABA (more complex rule, non-adjacent repetition; *N* = 30 trials) rule. For each experimental condition (shapes, upright faces, inverted faces), the 3 triplets were randomly selected from a set of 16 different ABB/ABA triplets that were generated combining 8 unique stimuli, 4 assigned to the ‘A’ group of images, and 4 assigned to the ‘B’ group of images. Each image within the triplet was presented in sequence from left-to-right, one at a time. The first shape was displayed alone on the left side of the screen for 330 ms, the second was added in the middle of the screen and displayed for 330 ms, and the last image appeared next to the others on the right side and displayed for 330 ms. A 500 ms blank was displayed between each of the three triplets. In total, the learning phase lasted 4500 ms. Twelve uninformative catch trials (ABC) were also presented to prevent anticipatory responses, and were not included in the analysis.

An attention-getter accompanied by a sound (500 ms) signaled the beginning of the testing phase. Four new exemplars of the stimuli (2 of them assigned to the ‘A’ group and 2 assigned to ‘B’ group of images) were employed to generate a new set of 4 different ABB/ABA triplets. In the test phase of each trial, a triplet was randomly selected from this new set and was displayed following the ABB or ABA rule. After each test triplet, the words ‘yes’ and ‘no’ appeared and remained on the screen until the participants responded by pressing a key on the keyboard. The left/right response key was counterbalanced across participants.

#### Corsi Block Tapping-Test (CBT)

The experimenter shows nine square blocks positioned on a board in front of the participant. The experimenter taps a sequence of blocks in a predetermined order at a rate of approximately one block per second. Immediately after, the participant was required to mimic the experimenter by tapping the blocks in the same order. The task starts with a small number of blocks (three blocks) and gradually increases in length up to nine blocks. For each length, three different sequences were administered. If the participants correctly succeeded, reproducing at least one of the three sequences with the same length, new sequence length increased by one item were presented. When the subject fails on two consecutive sequences of the same length the test ends. Participants’ span was defined by the length of the longest sequence correctly reproduced.

## Results

Reaction times and manual response accuracy (the mean accuracy of all experimental conditions for each group) were the dependent variables. Since reaction times were not normally distributed within each condition (*Ws* > 0.926, *ps* < 0.020), the RT data were logarithmically transformed. Moreover, a *d’* index was calculated as a measure of accuracy computed as the differences between the standard score (z-score) for the correct responses (hit) and false alarm rates (*d ′* = ZHit – ZFA; e.g., Macmillan & Creelman, 1990). The d’ index is particularly appropriate in tasks where a yes/no response is requested. It estimates the subject’s sensitivity to detect signals from noise as it measures the accuracy in detecting the congruent or incongruent stimulus by removing the effect of the high/low conservative criterion adopted by an individual participant to respond (for more explanations about this index see: Stanislaw & Todorov, 1999). Positive values indicated good performances, and a value of 0 indicated an inability to differentiate congruent and incongruent rules at the test. All the frequentist analyses were complemented with the same analysis performed by a Bayesian statistic in JASP 0.16.3 (JASP 2022) using the default Chaucy prior (*r* = 0.707). Using the Jasp formalism, the index next to the Bayes Factors (*BF*_*10*_) indicates that the null hypothesis (H0) is in the denominator and the alternative hypothesis (H1 in the numerator). Thus, *BF*_*10*_ is calculated as *p*(data|H1)/*p*(data|H0); *BF*_*10*_ > 10 is considered as a strong evidence for an effect, while 3 < *BF *_*10*_< 10 is considered a moderate evidence for an effect and *BF*_*10*_ < 3 indicated insufficient evidence to draw a conclusion for or against either hypothesis.

### Reaction times

**Fig. 2:**
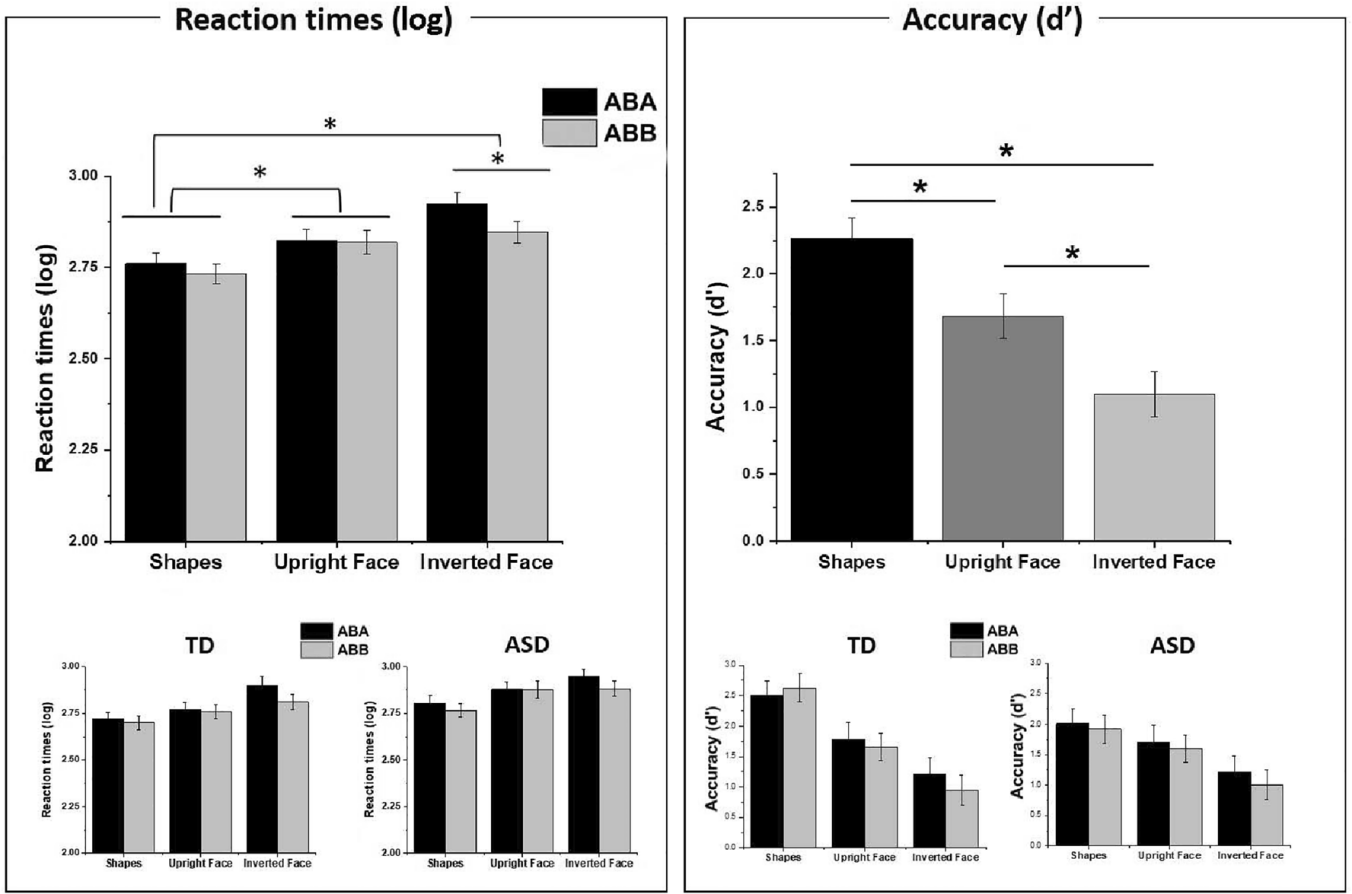
**a** Log-transformed reaction times (RTs) and **b** accuracy D-prime (d’) in detecting the ABB and ABA rules when shapes, upright faces and inverted faces were used as stimuli. In the lower panel, the RTs and d’ are shown separately for the ASD and TD groups

A repeated measure ANOVA (rmANOVA) was conducted on log-transformed reaction times (RTs) with Rule (ABB vs. ABA) and Condition (Shapes vs. Upright faces vs. Inverted faces) as within-subjects factors, and Group (TD vs. ASD) as between-subjects factors. The same analysis was complemented by Bayesian analysis with a default setting in null versus model comparison using the effect across matched model approach (see Mathôt, 2017; van den Bergh et al., 2020), while the interaction of factors was analyzed using *t* tests. The analysis revealed a main effect of Rule, *F* (1, 34) = 11.013, *p* = 0.002; *η*_*p*_^*2*^ = 0.245; *BF*_*10*_ = 4.38, a main effect of Condition, *F* (2,68) = 12.235, *p* < 0.001; *η*_*p*_^*2*^ = 0.265; *BF*_*10*_ > 100, and a Rule × Condition interaction, *F* (2,68) = 3.264, *p* < 0.044, *η*_p_^2^ = 0.088; *BF*_*10*_ = 2.34. The factor Group did not reach statistical significance (*p* > 0.070; *BF*_*10*_ = 1.28). No other interactions involving the Group factor were found (all *ps* > 0.625). Planned *t* tests (Bonferroni corrected with *α*/3 = 0.016) showed that RTs were faster in response to Shapes (*M* = 2.751; *SE* = 0.027) compared to both Upright faces (*M* = 2.825; *SE* = 0.175), *t*(35) = 3.005, *p* = 0.005, *d* = 0.148, 95% *CI* (− 0.845, 0.151), and Inverted faces (*M* = 2.885 ms; *SE* = 0.029), *t*(35) = 4.345, *p* < 0.001, *d* = 0.186, 95% *CI* (− 1.088, − 0.352). After Bonferroni correction, the differences between Upright faces (*M* = 2.825; *SE* = 0.029) and Inverted faces (*M* = 2.885; *SE* = 0.029) did not reach statistical significance, *t* (35) = 2.184, *p* = 0.036, *d* = 1.66; 95% *CI* (− 0.699, − 0.024). Further, participants were faster in response to ABB (*M* = 2.799; *SE* = 0.022) than to ABA rules (*M* = 2.836; *SE* = 0.023). The significant Condition × Rule interaction was further explored through a series of planned *t* tests (Bonferroni corrected, *α*/3 = 0.016). This analysis revealed that RTs were faster in response to the ABB (*M* = 2.847; *SE* = 0.030) compared to the ABA rule (*M* = 2.924; *SE* = 0.314) only in the Inverted face condition, *t* (35) = 3.409, *p* = 0.002, *d* = 0.136; 95% *CI* (0.212, 0.917) (Fig. 2). Two-tailed paired sample Bayesian *t* tests confirmed the results obtained from frequentist analysis, showing strong evidence for a difference between Shape and Inverted face (*BF *_*10*_> 100), moderate evidence for a difference between Shape and Upright face (*BF *_*10*_= 7.83), and weak differences between Upright and Inverted faces (*BF *_10_= 1.461). Moreover, the Bayesian analysis confirmed the results showing strong evidence for a difference between ABA and ABB in the Inverted face condition (*BF *_*10*_= 20.14).

### Accuracy (d’)

A rmANOVA on d’ index was conducted with Rule (ABB vs. ABA) and Condition (Shapes vs. Upright faces vs. Inverted faces) as within-subjects factors, and Group (TD vs. ASD) as between-subjects factors. The same analysis was complemented by Bayesian analysis on d’prime (see Mathôt, 2017; van den Bergh et al., 2020). The factor Group or interactions involving the Group factor did not reach statistical significance (all *ps* > 0.077). A significant main effect of Condition emerged from the analysis, *F* (2,68) = 31.359, *p* < 0.001, *η*_*p*_^*2*^ = 0.480, *BF *_*10*_> 100. Follow-up planned *t* tests (Bonferroni corrected, *α*/3 = 0.016) revealed that participants were more accurate in response to Shapes (*M* = 2.68; *SE* = 0.21) compared to Upright faces (*M* = 1.86; *SE* = 0.21), *t*(35) = 4.056, *p* < 0.001, *d* = 1.10; 95% *CI* (0.376, 1.117), and Inverted faces (*M* = 1.12; *SE* = 0.18), *t*(35) = 7.799, *p* < 0.001, *d* = 1.20, 95% *CI* (0.849, 1.740). Further, the accuracy was greater in response to Upright faces (*M* = 1.86; *SE* = 0.21) compared to Inverted faces (*M* = 1.12; *SE* = 0.18), *t* (35) = 4.805, *p* < 0.001, *d* = 0.919; 95% *CI* (0.420, 1.173) (Fig. 2). The two-tailed paired sample Bayesian *t* tests revealed moderate evidence for a difference between Shape and Inverted face (*BF *_*10*_> 3.33), and strong evidence for a difference between Shape and Upright face (*BF *_*10*_> 100), and between Upright and Inverted faces (*BF *_*10*_> 100).

### The relation between visual-spatial working memory and visual rule learning abilities

**Fig. 3:**
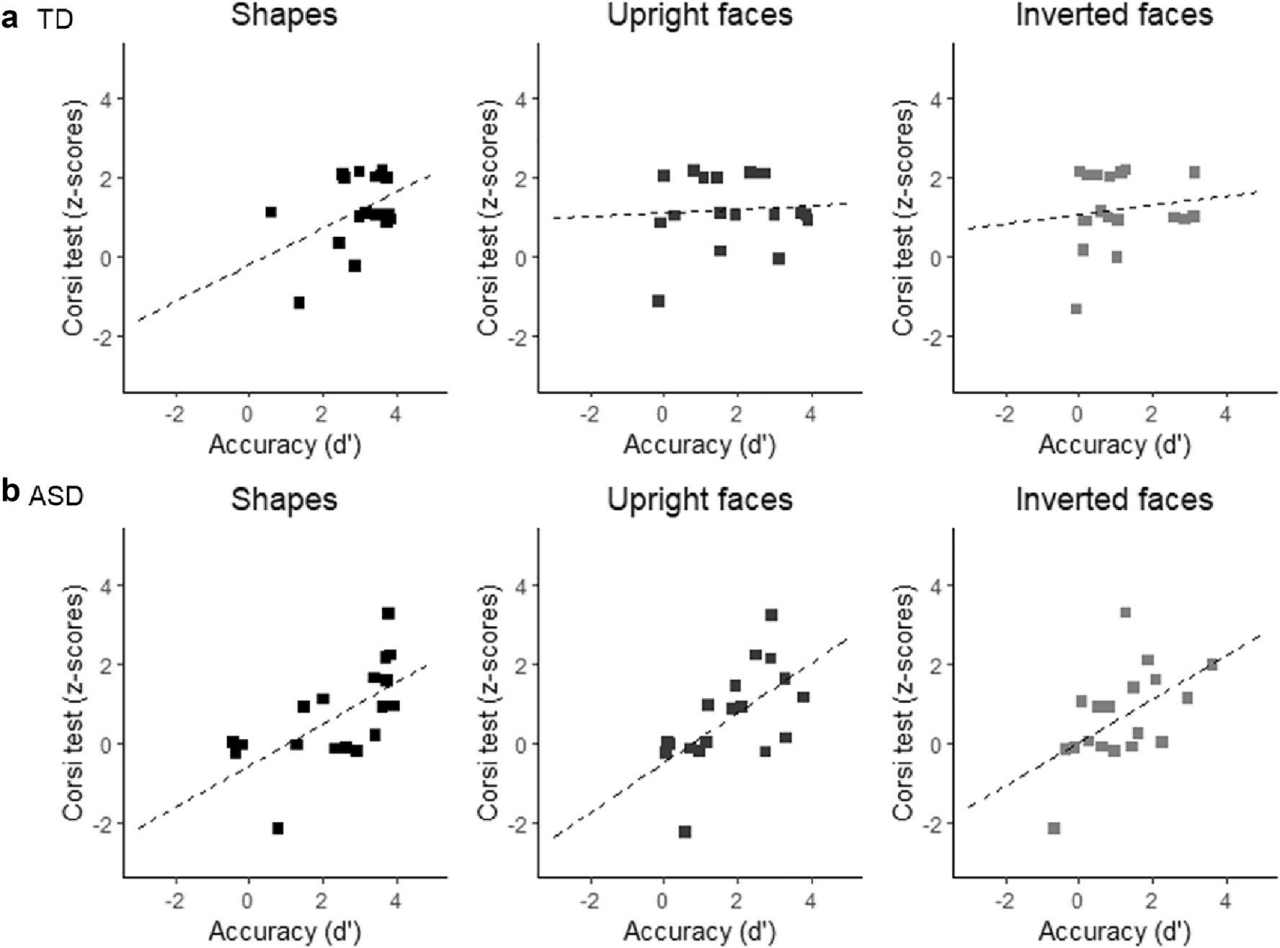
Correlations between accuracy D-prime (d’) and visual working memory scores (expressed as z-scores) in the three experimental conditions for the TD group (**a**) and the ASD group (**b**)

A frequentist and a Bayesian paired *t* test were first conducted on z-scores of the Corsi Block Tapping-test between ASD and TD groups, revealing no differences in the visual WM span, TD: *M* = 1.21, *SE* = 0.93; ASD: *M* = 0.65, *SE* = 1.22; *t* (34) = 1.250, *p* = 0.220, *d* =  − 0.513, 95% *CI* (− 1.174, 0.155), *BF *_*10*_= 0.801. Then, Pearson Correlations using a frequentist and Bayesian approach were conducted to explore within each experimental group (TD, ASD) the relation between d’ index and logRTs in response to the three conditions (Shapes, Upright faces, Inverted faces) with the z-scores of the Corsi Block Tapping-test. The analysis revealed a significant positive correlation between the Corsi score and the d’ index in response to Shapes, *r* = 0.653, *p* = 0.003, 95% *CI* (0.352, 0.782), *BF *_10_= 15.75, 95% *CI *(0.227, 0.839), Upright faces, *r* = 0.622, *p* = 0.006, 95% *CI *(0.038, 0.617), *BF *_*10*_= 9.76, 95% *CI *(0.183, 0.823), and Inverted faces, *r* = 0.525, *p* = 0.025, 95% *CI *(0.019, 0.605), *BF *_*10*_= 2.97, 95% *CI *(0.057, 0.770), only in the ASD group (Fig. 3). The analysis did not reveal significant correlations in the TD group (all *p*_*S*_ > 0.078). Finally, no correlations were found between the Corsi score and the logRTs in both groups (ASD: *ps* > 0.069; TD: *ps* > 0.385).

## Discussion

In the present study, we examined rule-learning abilities (RL) and the modulatory role of the social nature of the stimulus in the identification of high-order rules in adolescents affected by ASD and in a group of TD peers. Results demonstrate that ASD and TD groups displayed comparable RL performances independently from the social vs. non-social nature of the to-be-learned stimuli. However, this evidence is not fully supported by the Bayesian analysis, which does not allow for a conclusive exclusion of the hypothesis that the ASD group performed differently from the TD group. Even though previous findings have reported intact implicit learning processes related to stimulus-reward associations (Bottini, 2018) and statistical learning (Obeid et al., 2016) in ASD, further studies are needed to better understand the functioning of RL abilities in the ASD population.

The present data are not in line with our expectations regarding the possible effect of social stimuli in affecting RL abilities in ASD vs. TD participants. Indeed, stimulus characteristics have affected RL performance in the same way in both groups. ASD and TD peers were faster and more accurate in detecting and generalizing rules from shapes compared to upright and inverted faces. Shapes are characterized by only two perceptually salient features (i.e., color and form), and this might have facilitated the extraction of the rules. Differently, upright and inverted faces are perceptually more complex. The higher perceptual complexity of inverted faces might have heavily impacted the recognition of the high-order rules. Indeed, only when inverted faces were presented, both ASD and TD groups were significantly less accurate and slower to detect non-adjacent ABA rather than adjacent ABB rules. Converging literature suggests that ABB rule is easier to detect than non-adjacent ABA rules (Johnson et al., 2009), although it is not clear which is the mechanism that drives such differences in the decoding of adjacent repetition vs. non-adjacent repetition-based rules. Future studies should further explore the level of processing, i.e., featural or configural, used by ASD individuals to identify the relevant information provided by social vs. non-social stimuli within the ABB/ABA rule-like patterns. For example, an eye-tracker system could be used to examine visual scanning strategies during task execution in order to understand whether ASD adolescents have used local vs. holistic information in detecting differences in facial identities.

Finally, we found an association between visual–spatial WM and accuracy scores in rule recognition only in the ASD group. Specifically, an increase in memory span was associated with higher accuracy in detecting the rule in all stimulus conditions, i.e., with shapes, upright faces, and inverted faces. WM is considered a system that temporarily maintains, updates, and manipulates information required for learning (De Belder et al., 2015). Further, previous studies have reported deficits in both phonological and visual WM (for a review, see Habib et al., 2019; Unsworth et al., 2005) and an atypical developmental trajectory of WM in the ASD population (Wang et al., 2017). However, the results are controversial given that some evidence does not report deficits in visual WM in ASD (Lynn, Luna, & O'Hearn, 2022). Here, for example, we have not found differences in visual WM between TD and ASD adolescents (see, for example, Lynn et al., 2022; Ozonoff & Strayer, 2001). Thus, it is plausible to hypothesize that the current RL task might have been more demanding for ASD, and they might have used additional or alternative strategies that relied the most on visual WM resources compared to TD peers. For example, ASD adolescents might have employed over-used explicit strategies to perform the task. For instance, given that we provided spatial information in the task, it could be hypothesized that ASD individuals might have relied mostly on such spatial extra-cue to segment each triplet within the continuous flow of visual information and to link each item to a distinct position on the horizontal line (left, central, right). Future studies might investigate the role of space in RL processes in ASD populations by directly comparing conditions in which spatial information is provided or not.

The absence of differences between the behavioral performances of the ASD and the TD groups does not rule out the possibility that individuals with ASD might have extracted and generalized rule information using different cognitive and neural processes, a possibility not addressed in the current study. For example, it has been shown that ASD population has a similar behavioral performance on statistical learning tasks but shows different neural activation and processes underlying the behavioral performance (Jeste et al., 2015; Scott-Van Zeeland et al., 2010). Future studies might investigate RL abilities using neurophysiological measures that do not require explicit responses to rely on a more sensitive index to detect individual differences in RL abilities in the ASD population.

The lack of a direct comparison of IQ between groups might be considered as a limitation of the present study. Several studies have reported a relationship between IQ and implicit learning skills in both TD and ASD individuals (i.e., Jeste et al., 2014; Jones et al., 2018), thus it is possible that differences in IQ may, at least partially, account for the pattern of findings we obtained. Further studies are needed to explore the relationship between RL skills and IQ and its relation to autism symptoms.

To sum up, our results add important insights into the current literature. We have shown that visual learning of ABB and ABA rules in ASD adolescents, as in TD, is modulated by the nature and complexity of the stimulus, and to some degree, by the complexity of the rule. Moreover, we highlighted the fundamental role of WM on which ASD adolescents seem to rely more than TD peers to detect and generalize high-order rules. Along with previous evidence that showed difficulties in ASD adolescents in generalizing social rules to different social situations (e.g., Ozonoff & Miller, 1995), the present study suggests that poor generalization in the ASD group, without intellectual disabilities and with fluent language, might be specific to complex social interactions and might not be ascribed to a general impairment in the mechanism underlying rule generalization. Indeed, the generalization governing real social routines is far more complex than the generalization tested in the current task. However, to better understand the impact of contextual changes in RL processes, further studies might explore whether ASD adolescents are able to generalize a rule across different stimulus types, for example by presenting shapes in the learning phase and faces in the test phase or vice versa.

Overall, it could be hypothesized that difficulties in RL might arise when ASD individuals have to learn and generalize rules during more complex social routines. Training ASD individuals to use explicit strategies in RL tasks of increased difficulty, together with specific training aimed at improving their WM skills, might have a positive cascading impact on the development of more complex social skills, like the ones needed to extract and generalize very complex abstract structures characterizing human communication and interaction.
